# Stepping Together for Children After Trauma (ST-CT): Feasibility and Predictors of Outcome of a Parent-led, Therapist Assisted Treatment

**DOI:** 10.1007/s10802-024-01199-5

**Published:** 2024-05-13

**Authors:** Silje M. Ormhaug, Ingeborg Skjærvø, Gunvor M. Dyrdal, Else Merete Fagermoen, Kristin J. Haabrekke, Tine K. Jensen, Marie L. Knutsen, Anders Næss, Heidi Maria Päivärinne, Marianne Martinsen

**Affiliations:** 1 Norwegian Centre for Violence and Traumatic Stress Studies (NKVTS), Oslo, Norway; 2 Department of Health Sciences in Gjøvik, NTNU, Gjøvik, Norway; 3 Department of Psychology, University of Oslo, Oslo, Norway; 4 Faculty of Education, Innland Norway University of Applied Science, Hamar, Norway

**Keywords:** Parent-led treatment, Posttraumatic stress symptoms, Children, Feasibility, Stepped Care CBT for Children after Trauma (SC-CBT-CT)

## Abstract

Stepping Together for Children after Trauma (ST-CT) is the first step of the promising intervention Stepped Care CBT for Children after Trauma. In ST-CT, the task of leading treatment is partially shifted to the parents, and the child and parent work together to complete therapeutic tasks from a workbook with therapist supervision. We aimed to investigate the feasibility of ST-CT in Norwegian first line services and explore child factors predicting outcome. Eighty-two children (mean age 9.9 years, 56% girls) participated. Feasibility was defined by treatment completion, reductions of child posttraumatic stress symptoms (PTSS) mid- and post-treatment, and client treatment satisfaction. Predictors included child baseline PTSS, depressive symptoms, posttraumatic cognitions, externalizing symptoms, number of different traumatic events, and type of trauma. Results showed that rates of completion (78.0%) and response (81% of completers/59.8% intention-to-treat) were comparable to previous studies by the ST-CT developer. Overall treatment effect was *d* = 2.46 and client treatment satisfaction was high (mean score child: 8.3, parent: 9.0, on a scale from 0 – 10). Higher baseline PTSS and depressive symptoms predicted poorer outcome at both mid- and post-treatment, while more posttraumatic cognitions, and exposure to interpersonal trauma predicted poorer outcome at mid-treatment only. These associations were no longer significant in the fully adjusted models. In conclusion, ST-CT shows promise as an effective first line treatment in this new context, with two of three children responding to the treatment. Baseline PTSS, depression, post-traumatic cognitions and type of trauma may be related to outcomes and should be explored further. (Trial registration: ClinicalTrials.gov Identifier: NCT04073862. Retrospectively registered June 3rd 2019, first patient recruited May 19th 2019).

## Background

A substantial number of children are exposed to traumatic events every year (Hafstad et al., 2020; McLaughlin et al., 2013) and many develop impairing trauma-related symptoms (Alisic et al., 2014; Hiller et al., 2016). To date, several treatment models have documented effect in reducing children’s post-traumatic stress symptoms (PTSS) and related symptoms (see e.g., Bisson & Olff, 2021). However, currently the availability of evidence-based trauma treatments is limited – in particular in low- and middle income countries (Yatham et al., 2018), but also in high-income countries (Bringewatt & Gershoff, 2010; Schweer-Collins & Lanier, 2021). This will potentially lead to post-trauma symptoms developing into severe mental health issues over time (Hiller et al., 2016). Identifying effective and accessible interventions allowing children to regain a healthy development and function is therefore crucial (Lewis et al., 2019). Stepped care models can be a way to reduce the gap between the need for treatment and the access to evidence based care (McDermott & Cobham, 2014). Typically, stepped care models involve task shifting where in the first, lower intensity step, the therapeutic tasks are delegated to lay persons. This reduces the need for therapeutic resources, shortens wait time, and allows for treatment provision at a lower cost. In models where the tasks of leading the treatment are shifted to a parent, treatment barriers can be reduced, including time spent traveling to see the therapist, taking time off from work and the need for childcare. Further, the parent’s wish to help and solve their children’s problems themselves can be met (Thurston & Phares, 2008) and parenting skills strengthened (Salloum et al., 2014). Studies on parent-led treatments for child anxiety have shown good results (Creswell et al., 2022), however including parents as co-therapists is still a new and unfamiliar mode of treatment for many therapists. The current study investigates the feasibility of one such innovative model for PTSS specifically seeking to reduce the described gaps and simultaneously strengthen parents’ capacity to help their own children heal.

*Stepped Care CBT for Children after Trauma* (former Stepped Care TF-CBT; Salloum et al., 2014, 2022a, b) is comprised of two steps. In Step One, *Stepping Together for Children after Trauma* (ST-CT) the responsibility of leading the treatment is shared with the parent. In this step the child and parent work together to complete therapeutic tasks defined in a workbook. The workbook is based on the Preschool PTSD Treatment (Scheeringa, 2016; Scheeringa et al., 2011) and builds on the empirically supported principles of cognitive behavioral therapy (CBT) for posttraumatic stress (e.g., Cohen et al., 2004; Scheeringa et al., 2011). While most parenting programs are directed towards improving parenting skills, the current model aims to directly reduce the child’s trauma symptoms through teaching the parent to help the child complete the therapeutic tasks. This includes learning relaxation and emotion regulation skills, and tasks to enhance trauma memory exposure and processing. The children who do not improve sufficiently to meet the responder criteria after completing the active treatment phase (normally 6 – 9 weeks), or are unable to complete the treatment at home, are stepped up to Step Two, which in the original model consists of therapist-led *Trauma-Focused CBT* (Cohen et al., 2017). Those who, on the other hand, show sufficient improvement continue to a maintenance phase of 6 weeks to make sure the positive development is maintained (for further details of the model, see Method section). In this paper we investigate the feasibility of ST-CT (Step One) as a stand-alone treatment in the first-line municipality services in Norway.

So far, studies of the two-step SC-CBT-CT model have shown encouraging results. Results from three randomized trials (*n* = 33, *n* = 53, and *n* = 183) show that SC-CBT-CT is non-inferior in reducing PTSS compared to standard, therapist-led TF-CBT for children between 3–12 years, and that costs are between 38–62% lower (Salloum et al., 2017, 2016a, b, 2022a, b). Further, studies on SC-CBT-CT indicate that both children and parents are satisfied with the model (Fagermoen et al., 2023a; Muster et al., 2022; Salloum et al., 2015a, b, 2016a, b).

To date, this two-step intervention has been tested by the model developers on US samples as a unified model. The parent-led first step is a new and innovative mode of treatment that has not been tested as a stand-alone intervention before, and it is new to therapists in Norway. If proven helpful, this intervention can contribute to reduce the overall cost-burden of health care services. Also, since parenting practices vary across cultural settings it is not given that a model that is suitable for one context fits in a new one. Before expensive effectiveness testing and implementation processes can be recommended, more knowledge of the parent-led model’s feasibility in different service levels as well as different cultural and economic settings is useful. This includes limited-effectiveness testing of the model (e.g., assessing symptoms pre-post treatment), investigating the acceptability for children and caregivers, and evaluating the potential for expansion by comparing results to previous findings (Bowen et al., 2009). Further, since ST-CT is still a fairly new model, knowledge of tailoring variables that can help therapists choose the most suitable treatment level for each child is scarce (Salloum et al., 2022a). Thus, feasibility trials in new settings can also contribute to important knowledge such as which children are more likely to benefit from the low-threshold parent-led treatment and who should be allocated directly to therapist-led treatment.

Available research on the parent-led ST-CT model indicate that parent variables such as depression, Latino/Hispanic background, lower levels of education, higher level of emotional reactions to their child’s trauma, and higher levels of social support predict poorer outcome of the parent-led ST-CT model (Salloum et al., 2022a; Fagermoen et al., 2023a). There is little knowledge, however, of child characteristics related to outcome. The one study that has investigated this (*n* = 62) found that children with severe anger outbursts were less likely to respond to parent-led ST-CT, whereas variables such as age, gender, baseline PTSS, type and number of trauma exposure were not related to outcome (Salloum et al., 2022a). These findings are in line with the therapist-led child trauma literature. Results from a recent individual participant data meta-analysis (*n* = 1,686) found that the efficacy of a variety of therapist-led cognitive behavioral therapies with a trauma focus was not moderated by child age, gender, or trauma characteristics (de Haan et al., 2023). There was a somewhat lower effect associated with higher levels of externalizing symptoms, whereas higher levels of initial PTSS, depression and anxiety were associated with amplified treatment effects. Given that factors related to treatment outcomes may differ in parent-led treatment compared to therapist-led treatment, and that the one study on ST-CT included a relatively low *n,* more studies are needed to identify child tailoring variables in parent-led treatments.

## Current Study

The aims of this study were two-fold. First, we investigated the feasibility of the parent-led ST-CT as a stand-alone treatment in Norway. This included limited-effectiveness testing (investigating children’s symptom development pre – post treatment, dropout and response rates), investigating the model’s acceptability (child and caregiver reported satisfaction with the treatment), and potential expansion by comparing the results from the current study with the parent-led ST-CT in the two previous studies by the developer that included children in the same age range as the current study (7–12; Salloum et al., 2017, 2022a, b).

Secondly, we aimed to learn more about child tailoring variables. More specifically we wanted to examine whether child factors such as baseline PTSS, depressive symptoms, posttraumatic cognitions, externalizing symptoms, and number and type of trauma exposure (non-interpersonal- vs interpersonal trauma) were associated with outcome of the parent-led ST-CT. Based on the currently available research it was hypothesized that higher levels of externalizing problems would predict higher levels of PTSS at T2 (after the active treatment phase), and at T3 (after the maintenance phase), but that there would be no significant effect of baseline PTSS, depression, posttraumatic cognitions, exposure to a higher number of different traumatic events, or exposure to interpersonal trauma.

## Method

### Sample

Children with PTSS and their caregivers participated in an open trial investigating the feasibility of parent-led ST-CT in 11 low-threshold services that provide short-term treatment for children in Norway. The final sample consisted of 82 child-caregiver dyads, with 56% girls and child average age of 9.9 years (*SD* 1.45, range 7–12).

Child inclusion criteria were: 1) age 7 to 12 years; 2) at least four weeks since exposure to one or more potentially traumatic events according to DSM-5 (American Psychiatric Association, 2013) after age 3; and 3) experiencing at least five DSM-5-defined PTSS, including at least one symptom of both re-experiencing and avoidance. Participants did not need to meet full criteria for any of the DSM-5 trauma disorder diagnoses. Exclusion criteria: 1) the participating caregiver was the perpetrator, or the child was living with a perpetrator; 2) indications of child or parent psychotic symptoms, cognitive disability, active suicidal thoughts, or other conditions that could limit the child’s or parent’s ability to complete the workbook; 3) the need for an interpreter; 4) untreated parental substance abuse; or 5) the child received concurrent trauma-focused psychotherapy. See Skjærvø et al. (In review), for more details on the study.

### Treatment

**Fig. 1:**
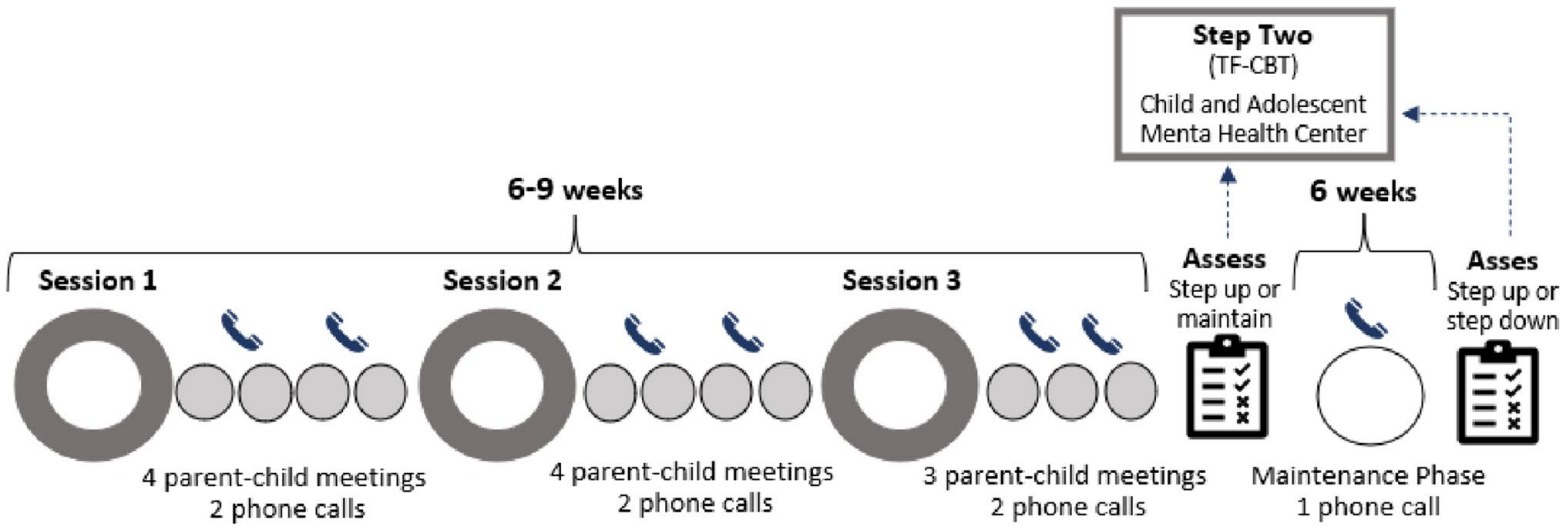
Overview of the ST-CT model in Norway

The parent-led Stepping Together-CT includes five main components: psychoeducation, stabilization skills, trauma narrative, in-vivo exposures, and consolidation. The caregiver and child work together to complete the tasks in the workbook, *Stepping Together* (Salloum et al., 2010). The tasks in the workbook focus on building coping skills and having the child complete trauma-focused exposures including developing a trauma narrative and in vivo exposure to trauma reminders. There are 11 parent-led meetings with the child at home, and three to five therapist-led sessions at the therapists’ office. In between the in-office sessions, the therapist schedules short, weekly consultation calls (10–15 min) with the parents to provide guidance and support as they work through the at-home meetings with the child. At the first meeting the therapist conducts a global rating of the child’s symptom severity (CGI-S). To monitor symptom development during the treatment and make decisions regarding the potential need for a child to be stepped up, a rating of improvement (CGI-I) is conducted by the therapist, child, and caregiver at each session, and by the caregiver during each phone session. After completion of the workbook (normally 6–9 weeks), the child’s symptoms are assessed (T2). Based on the T2 symptom assessment, children demonstrating sufficient response to treatment (see response criteria, below) are transitioned into the 6- weeks maintenance phase. This phase includes weekly at-home meetings where child and parent focus on maintaining the child’s coping skills and doing positive activities together, and the parent receives one phone call from the therapist. After the maintenance phase the final assessment (T3) is completed. If the child still meets the responder-criteria at T3, the treatment is considered complete, and the child and caregiver have a final short session with the therapist to conclude the treatment. For an overview of the model, see Fig. 1.

An adaptation of the model in the Norway was that whilst Step One was provided in the municipal first line services, Step Two was provided in the second line Child and Adolescent Mental Health Services (CAMHS). This implied that for children not meeting responder-criteria, the responsibility for the treatment was transferred from the municipal service level to the CAMHS, who provide TF-CBT.

#### Response Criteria

A child was defined a responder in line with Salloum et al. (2022a, b), i.e., if 1) he/she reported ≤ 4 symptoms of posttraumatic stress with a score of 2 or 3 (i.e., experiencing the symptom “*half the time*” or “*almost always*”) on the Child and Adolescent Trauma Screen 2 (CATS 2; Sachser et al., 2022), and 2) the therapists rated the child’s global improvement (Clinical Global Impression – Improvement [CGI-I]; Guy, 1976) to be 3 or lower (i.e. *improved*, *much improved* or *symptom* *free*. See description below).

#### Fidelity

All therapist-led sessions were audiotaped, and based on a checklist developed by Dr. Salloum, fidelity was assessed by trained supervisors. Model fidelity was high (above 95%), agreement between the ratings was above 95%, and all cases were approved.

#### Therapists and Training

In the current study, 19 therapists were trained and provided Stepping together CT. See Skjærvø et al. (In review) for more details about the training.

## Procedure

The study was performed in line with the principles of the Declaration of Helsinki. Approval was granted by the Regional Ethics Committee prior to start (REK 2018/771).

Children and their caregivers were consecutively recruited by the therapists working in the municipalities. The therapists screened the children for potentially traumatizing events with CATS-2 and assessed the eligibility criteria for participation. To limit selection bias, therapists were asked to invite all the families of which the child fulfilled the inclusion criteria, and not make judgements based on their own expectations of suitability. When in doubt, therapists could consult supervisors in the project group. Both children and caregivers were informed that participation was voluntary, and that non-participation would not affect access to therapy. Caregivers gave written consent while children confirmed their consent to participate orally to the therapist.

Both the child and the participating parent completed assessments pre-treatment (T1), after completion of the active treatment phase, mid-treatment (T2), and after the maintenance phase, post-treatment (T3). The child assessments were completed on a tablet, with the therapist in the room to clarify misunderstandings due to differing levels of reading competencies. Caregivers self-completed their assessments on a tablet. All data was encrypted and stored safely at the University of Oslo.

## Measures

All questionnaires were piloted on children and caregivers to assess time to complete and receive input on the measures.

### Potentially Traumatizing Events, Symptoms of Post-Traumatic Stress, and Functional Impairment

The Child and Adolescent Trauma Screen 2 (CATS-2; child and caregiver proxy-version) was used to assess the child’s exposure to potentially traumatizing events and related symptoms. CATS-2 accommodates for the adjustments in DSM-5 and ICD-11 and is validated for children aged 7–17 (Sachser et al., 2022). CATS-2 addresses life-time exposure to 15 potentially stressful or frightening experiences (*yes/no*), whereof natural disaster, severe accident, sudden death of a caregiver/close person and scary medical treatment were defined as non-interpersonal trauma, and child abuse, witnessing violence, being threatened, or attacked, forced sexual activities, and severe bullying were defined as interpersonal trauma. Further, CATS-2 assesses level of post-traumatic stress symptoms during the last 4 weeks (20 items: intrusions/re-experiencing, avoidance, negative changes in cognition/mood and hyperarousal). Items are scored from 0 (*never*) to 3 (*almost always*), giving a sum score (range 0–60). A score of 25 has been found to be the cut-off for probable PTSD (ibid). Five items assess interference of symptoms on psychosocial functioning in the last 4 weeks (*yes/no*). Internal consistency of the symptom scale was acceptable (α = 0.79).

### Clinical Severity and Improvement

The Clinical Global Impression Severity (CGI-S) and Improvement (CGI-I) scales were used to assess clinical severity and improvement before and during treatment. These are two 1-item global assessments of status and function (CGI-S) and subsequent change in function from session to session (CGI-I). The CGI-S is scored on a 7-point scale from 0 (*no symptoms*) to 6 (*extremely ill*) (Busner & Targum, 2007; Guy, 1976), and the CGI-I is scored on an 8-point scale from 1 (*no symptoms*) to 8 (*very much worse*) (Salloum et al., 2015a, b). Both the CGI-S and CGI-I have been found to be easily understandable and useful for non-researcher clinicians (Busner & Targum, 2007).

### Child Posttraumatic Cognitions

Child posttraumatic cognitions were assessed with the Children's Post-Traumatic Cognitions Inventory Short (CPTCI-S). The CPTCI-S is a short version of the full CPTCI 25 item measure, assessing negative thoughts children can develop following trauma (McKinnon et al., 2016; Meiser-Stedman et al., 2009). The items cover the two dimensions “feeble person in a scary world” and “permanent and damaging change” and items are scored on a scale from 1 (*do not agree at all*) to 4 (*agree a lot*) and summed (range 10–40). A score ranging from 16–18 is indicative of clinically significant levels of cognitions. The CPTCI-S has been shown to have moderate-to-high test–retest reliability (*r* = 0.78; McKinnon et al., 2016) and internal consistency of the full scale in this sample was good (α = 0.83).

### Child Depressive Symptoms

The Short Moods and Feeling Questionnaire (SMFQ) was used to assess child depressive symptoms. This questionnaire consists of 13 statements where children self-report symptoms of depression in the last two weeks. The scale has been validated internationally among children aged 7–11 years (Angold et al., 1995; Sharp et al., 2006). The items are scored 0 (*not true*), 1 (*sometimes true*) and 2 (*true*) and summed (range 0–26, clinical cut-off ≥ 12)﻿. The internal consistency in the current sample was good (α = 0.88).

### Externalizing Behavior

To assess externalizing behaviors, the validated externalizing behavior subscale (fighting, teasing) of the Pediatric Symptom Checklist (PSC-17) (Gardner et al., 2007) was used. The 7 items are scored from 0 (*never*) to 2 (*often*) and summed (range 0–14, clinical cut-off ≥ 7). The PSC-17 externalizing subscale has been found to perform equally well as longer questionnaires (Gardner et al., 2007; Parker et al., 2019), and internal consistency in the current sample was acceptable (α = 0.72).

### Child and Caregiver Satisfaction with the Treatment

The children’s treatment satisfaction was measured with 3 questions about the therapy experience (Ormhaug et al., 2015): I liked coming to the therapist; Going to the clinic helped me with my problems; If I were ever having problems again, I would want to come back to this clinic. The three items are scored from 1 (*all the time*) to 4 (*not at all*) and summed (range 3 – 12), and internal consistency in the sample was good (α = 0.82). Parent’s satisfaction was measured with the Client Satisfaction Questionnaire (CSQ-8). This is the most used and validated assessment of caregivers’ satisfaction with a child’s treatment (Attkisson & Zwick, 1982; Larsen et al., 1979). The 8 items are scored from 1 (*poor*) to 4 (*excellent*) and summed (range 8 – 32), and internal consistency in this sample was excellent (α = 0.92). Assessments were conducted after treatment-completion, or when the child left the treatment (stepped up or dropped out).

## Data Analytic Plan

Characteristics of the child and parent samples were investigated with descriptive statistics, and inspections of potential outliers were conducted. Magnitude of changes in symptoms from pre to post treatment was investigated with paired samples *t*-tests, and effect sizes were based on Cohen’s *d*. Values of 0.2 were considered small effect, 0.5 a medium effect, and values above 0.8 were considered as large effects (Cohen, 2013). Comparisons between the current study and the studies by Salloum et al., 2017, 2022a﻿, b were conducted with chi square tests with Monte Carlo simulations (10,000) and *t*-tests. To investigate potential predictors of response, baseline levels of PTSS, clinical severity, post-traumatic cognitions, depression, and externalizing problems were compared between responders and non-responders with *t*-tests. Further, linear mixed models with children grouped within therapists were first run in an attempt to account for the nested nature of the data. However, these analyses resulted in unstable models, so the predictor analyses were conducted with single level linear regression analyses. Unadjusted models with single variables were run first to investigate prediction of baseline variables on child PTSS mid-treatment (T2) and post-treatment (T3). Subsequently, all variables were entered together in adjusted models. Pre-defined hypotheses were registered May 2019 based on the then existing knowledge (see ClinicalTrials.gov Identifier NCT04073862), however recent results from Salloum et al. (2022a, b) and de Haan et al. (2023) provided arguments for changing the hypotheses as presented in the introduction. Descriptive and pre-post analyses were conducted in SPSS (IBM, 2021), and linear regressions were run in R with the lm-package (Hornik, 2018).

### Missing Data

As inherent in the stepped care model, some children were stepped up to receive more therapist-intensive treatment at the CAMHS before completion of the active ST-CT treatment phase (T2). We do not have follow-up data on the child/caregiver dyads who were stepped up or dropped out, and therefore, the *n* varies at the different assessment points. In addition, 9 children did not complete the Functional impairment scale at T1. The treatment satisfaction scale data was missing for 4 out of 7 (57%) of those who were stepped up at T2 and for 1 of the 55 with scores at T3 (1.8%). To assess selective participation, missing data at mid-treatment was regressed on scores of child PTSS at pre-treatment, and missing data at post-treatment was regressed on scores of PTSS at pre-treatment and mid-treatment separately. Results indicated that a higher level of PTSS at pre-treatment was not significantly associated with missing data mid-treatment (*OR* = 0.95, *p* = 0.105). However, a higher level of PTSS pre-treatment and mid-treatment was significantly associated with a lower likelihood of having data post-treatment (*OR* = 0.90, *p* = 0.003, and *OR* = 0.67, *p* = 0.006). This was expected since stepping up was a decision based on high symptom-scores and shows that missingness must be assumed to be not at random (MNAR).

## Results

### Feasibility of ST-CT

#### Child Trauma Exposure and Baseline Symptoms

The sample was highly exposed to trauma, with the children reporting exposure to 3.8 (*SD* 2.1) different potentially traumatizing events, and the majority had an interpersonal trauma as their index trauma (68.3%). Overall, the children in the sample reported high levels of posttraumatic stress at baseline with 61 of the children (74.4%) scoring above the clinical range. Also, levels of posttraumatic cognitions were on average high (73.2% above clinical range), whereas the children presented with relatively lower levels of depressive symptoms (34.1% above clinical range) and symptoms of externalizing behaviors (11.0% above clinical range). For more details, see Table 1.

**Table 1:** Child sample characteristics

Characteristic	*n*(%)	Mean (*SD*)
*Child Background*		
Born outside Norway	3 (3.7)	
*Living situation*		
With both parents	41 (50.0)	
With one parent/ co-habiting	30 (36.6)	
Other (foster care, unknown)	11 (13.4)	
*Index trauma*		
Domestic violence	23 (28.0)	
Severe bullying	14 (17.1)	
Violence outside the family	14 (17.1)	
Death of significant person	7 (8.5)	
Scary medical procedure	6 (7.3)	
Sexual abuse	5 (6.1)	
Frightening separation from parent^a^	5 (6.1)	
Severe accident	4 (4.9)	
Natural disaster	3 (3.7)	
Other	1 (0.8)	
*Above clinical cutoff*^b^	61 (74.4)	
Probable PTSD	23 (28.0)	
Probable CPTSD	6 (7.3)	
Baseline PTSS		29.0 (7.7)
Baseline depressive symptoms^c^		10.0 (5.9)
Baseline PTC^d^		22.6 (6.2)
Baseline externalizing symptoms^e^		2.9 (2.4)

^a^Includes events such as getting lost in the woods, witnessing parent being arrested

^b^Assessed with the Child and Adolescent Trauma Scale 2 (CATS-2): range 0–60, clinical cut-off ≥ 25; PTSD = Posttraumatic stress disorder, CPTSD = Complex PTSD, PTSS = Posttraumatic stress symptoms, PTC = posttraumatic cognitions

^c^Assessed with the Short Mood and Feelings Questionnaire (SMFQ): range 0–26

^d^Assessed with the Child Posttraumatic Cognitions Index Short (CPTCI-S): range 10–40

^e^Assessed with the Pediatric Symptom Checklist (PSC-17), externalizing subscale; range 0–14

#### Child Dropout and Response Rate

**Fig. 2:**
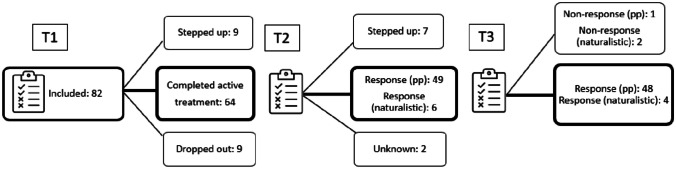
Participant Flowchart

Of the 82 included families, 64 (78.0%) completed the workbook and were assessed again at T2. Nine families dropped out, and nine were stepped up during the active treatment phase. Of the 64 that did complete the active treatment phase (T2), 49 (76.5%) met the responder criteria as defined by the protocol (per protocol sample). In addition, the therapists chose to send six of the families to the maintenance phase, based on clinical judgement and discussions with the families (naturalistic sample). After maintenance (T3), 52 (81.3%) of those who completed the active treatment phase did not need further trauma treatment, 48 of the children in the per-protocol sample and an extra 4 of the 6 in the naturalistic sample. Altogether 59.8% (per protocol)/63.4% (naturalistic) of the total sample met responder criteria at T3. (See Fig. 2).

#### Treatment Effect

On average, the children reported significantly lower symptom levels post treatment. After completion of the active treatment phase (T2, *n* = 63), the mean score was 13.6 (*SD* 9.7, range 0–46), which is in the normal range, and only 8 children (8.9%) scored above clinical cutoff for PTSD. This reduction equals a Cohen’s *d* of 1.52 (95CI: 1.16–1.88). For those who responded and were sent to the maintenance phase (*n* = 55), the mean symptom score was reduced from 11.0 (*SD* 6.4, range 0–29) to 7.7 (*SD* 5.6, range 0–20) post-treatment, Cohen’s *d* = 0.67 (95CI: 0.38–0.96), and none of the children were above the clinical cutoff. Overall change from pre- to post treatment vas Cohen’s *d* = 2.46 (95CI: 1.92–2.99).

Child reports of functional impairment decreased from 3.3 (*SD* 1.4, range 0–5, *n* = 73) at baseline to 1.4 (*SD* 1.5, range 0–5, *n* = 60) mid treatment, Cohen’s *d* = 1.17 (95CI = 0.82–1.50). After treatment, the mean score was reduced to 0.7 (*SD* = 1.2, range 0–5, *n* = 51), Cohen’s *d* mid–post = 0.31 (95CI = 0.02 – 0.60). Overall change pre–post was Cohen’s *d* = 1.40 (95CI: 0.98–1.81).

#### Client Satisfaction

Overall, both children and parents reported high levels of satisfaction with the treatment. Mean child score (*n* = 58) was 10.0 (Median 11.0, *SD* 2.0, range 4–12) and mean parent score (*n* = 58) was 28.8 (Median 30.0, *SD* 4.1, range 13–32). These scores equal 8.3 and 9.0 on a converted scale from 1–10, respectively.

#### Comparisons with Previous Studies

Analyses showed that overall, the current results are comparable to the outcomes of the parent-led Step One in the two previous studies by Salloum et al. (2017, 2022a, b). There were no statistically significant differences in terms of response rates, step-ups during the active phase, or treatment satisfaction (all *p-*values > 0.050), but the current study had lower dropout-rates compared to Salloum et al. (2022a, b). The study samples were comparable in terms of child baseline PTSS and number of children reporting interpersonal trauma as index trauma. However, child age was significantly higher in the current study compared to Salloum et al. (2022a, b), and a significantly higher number of children reported exposure to more than one traumatic event (88%) in the current study as compared to Salloum et al. (2022a, b) (55%) and Salloum et al. (2017) (55%). For more details regarding the comparisons, see Table 2.

**Table 2:** Findings compared to Salloum et al. 2017, 2022a, b

	**Ormhaug et al.**	Salloum et al. (2017)	***p-*value**	Salloum et al. (2022a, b**)**	***p-*value**
*N* participants in ST-CT condition	82	22		91	
Age, mean (*SD,* range)	9.9 (1.45, 7–12)	9.6 (1.5, 8–12)	0.385	8.3 (2.4, 4–12)	**< 0.001**
Girls, *n* (%)	46 (56.1)	13 (59.1)	0.813	48 (52.8)	0.754
Baseline PTSS (0–100), mean (*SD*)	48.3 (12.8)^a^	42.7 (18.1)^b^	0.189	46.8 (11.3)^c^	0.403
More than one traumatic event, *n* (%)	72 (88.2)	12 (54.5)	**0.002**	50 (54.9)	**< 0.001**
Index trauma interpersonal, *n* (%)^d^	56 (68.3)	19 (86.4)	0.116	65 (71.4)	0.738
Dropout, *n* (%)	9 (11.0)	4 (18.2)	0.468	21 (23.0)	**0.045**
Stepped up during active phase,* n* (%)	9 (11.0)	3 (13.6)	1.00	14 (15.4)	0.500
Responders T2 ITT, *n* (%)	49/82 (59.8)^e^	14/22 (63.6)	0.804	43/91 (47.3)	0.122
Responders T2 completers, *n* (%)	49/64 (76.6)^f^	14/17 (82.4)	0.751	43/61 (70.5)	0.539
Parent’s satisfaction, mean (*SD*)^g^	28.8 (4.1)	30.8 (3.3)	0.056	29.8 (3.1)	0.136

Effect size Cohen’s *d*					
T1-T2	1.52	1.47		1.02	
T1-T3	2.46	1.39		1.36	

P-values were tested with Monte Carlo 10,000 replications and *t*-tests

^a^Assessed with the Child and Adolescent Trauma Scale 2 (CATS-2): range 0–60, clinical range ≥ 25 / converted 0–100: clinical range ≥ 42

^b^Assessed with the University of California Los Angeles PTSD Index (UCLA PTSD Index): range 0–68, ≥ 38 = clinical range/ converted 0 – 100: clinical range ≥ 56

^c^Assessed with the Trauma Symptom Check-list for Young Children (TSCYC-PTS): range 27–108, clinical range ≥ 40 / converted 0–100: clinical range ≥ 37

^d^Reported events defined as interpersonal trauma: Sexual abuse, domestic violence, physical abuse, community violence, severe bullying, witnessing crime, parental arrest, kidnapping

^e^Per protocol sample. Corresponding results in the naturalistic sample is 63.43%

^f^Per protocol sample. Corresponding results in the naturalistic sample is 85.93%; ITT = intention to treat

^g^Assessed with the Client Satisfaction Questionnaire (CSQ-8). *N* = 58 in masked:* n* = 15 in Salloum 2017; n = 61 in Salloum 2022

## Predictors of Outcome

Analyses showed that children that were stepped up after completing the active treatment phase also reported higher baseline levels of PTSS (mean difference 8.8, *t*[1,61] = 4.0, *p* < 0.001), higher levels of depressive symptoms (mean difference 4.9, *t*[1, 57] = 2.7, *p* = 0.010), higher levels of posttraumatic cognitions (mean difference 4.2, *t*[1, 57] = 2.0, *p* = 0.045), and lower levels of externalizing symptoms (mean difference –1.4, *t*[1.58] = -2.8, *p* = 0.008). These results are mostly mirrored in the predictor analyses showing that baseline PTSS (Est. 0.52, *p* < 0.001), depressive symptoms (Est. 0.72, *p* < 0.001), posttraumatic cognitions (Est. 0.51, *p* = 0.009) and exposure to interpersonal trauma (Est. 5.91, *p* = 0.021) predicted higher PTSS levels after completion of the active treatment phase (T2) in the unadjusted models. After maintenance (T3), baseline PTSS (Est. 0.24, *p* = 0.013) and depressive symptoms (Est. 0.30, *p* = 0.033) continued to predict higher levels of PTSS. None of these findings remained significant in the fully adjusted models that included baseline PTSS, depression, posttraumatic cognitions, externalizing behaviors, type, or frequency of trauma exposure (all *p*-values > 0.050). For all results of the predictor analyses, see Table 3.

**Table 3:** Predictors of child PTSS at T2 and T3 – unadjusted and adjusted models

**Outcome: PTSS T2**	**Unadjusted**			**Adjusted**		
**Variable**	**Est**	***p***	**95 CI**	**Est**	***p***	**95 CI**
Sex: girl	-0.48	0.847	-5.40, 4.45	-1.93	0.449	-7.01, 3.15
Age	0.28	0.735	-1.38, 1.94	-0.61	0.499	-2.40, 1.19
Baseline PTSS^a^	0.52	**< 0.001**	0.24, 0.79	0.29	0.200	-0.16, 0.73
Baseline depression^b^	0.72	**< 0.001**	0.33, 1.12	0.64	0.070	-0.05, 1.34
Baseline PTC^c^	0.51	**0.009**	0.13, 0.88	-0.08	0.776	-0.65, 0.49
Baseline externalizing^d^	-0.47	0.369	-1.58, 0.64	-1.01	0.163	-2.45, 0.43
Total number traumas^a^	0.60	0.335	-0.64, 1.83	0.29	0.640	-0.95, 1.53
Interpersonal trauma^e^	5.91	**0.021**	0.91, 10.90	3.12	0.281	-2.64, 8.88

**Outcome: PTSS T3**	**Unadjusted**			**Adjusted**		
**Variable**	**Est**	***p***	**95 CI**	**Est**	***p***	**95 CI**
Sex: girl	-0.04	0.982	-3.10, 3.02	-1.73	0.306	-5.13, 1.65
Age	-0.35	0.504	-1.38, 0.69	-0.38	0.579	-1.55, 0.80
Baseline PTSS^a^	0.24	**0.013**	0.05, 0.43	0.11	0.442	-0.18, 0.40
Baseline depression^b^	0.30	**0.033**	0.02, 0.58	0.21	0.402	-0.29, 0.70
Baseline PTC^c^	0.21	0.084	-0.03, 0.46	-0.00	0.979	-0.37, 0.36
Baseline externalizing^d^	-0.24	0.471	-0.89, 0.42	-0.57	0.210	-1.47, 0.33
Total number traumas^a^	0.35	0.359	-0.42, 1.12	0.36	0.411	-0.47, 1.19
Interpersonal trauma^e^	0.24	0.878	-2.90, 3.39	-0.69	0.699	-4.26, 2.88

^a^Assessed with the Child and Adolescent Trauma Scale

^b^(CATS-2): range 0—60, clinical cut-off ≥ 25; PTSD = Posttraumatic stress disorder, CPTSD = Complex PTSD, PTSS = Posttraumatic stress symptoms, PTC = posttraumatic cognitions

^c^Assessed with the Short Mood and Feelings Questionnaire (SMFQ): range 0—26

^d^Assessed with the Child Posttraumatic Cognitions Index Short (CPTCI-S): range 10—40

^e^Assessed with the Pediatric Symptom Checklist (PSC-17), externalizing subscale; range 0—14

## Discussion

Parent-led Stepping Together CT is a promising treatment for traumatized children that can help bridge the gap between the need for and access to care by addressing treatment barriers and reducing the need for therapist resources. In this study, which is the first outside the U.S., results indicate that the parent-led model is feasible in a Norwegian setting as it both seems to be as effective as in the original setting and is well accepted by children and parents. However, treatment effect might be moderated by some child baseline characteristics that warrant further investigation.

The first aim of the study was to evaluate the feasibility of the parent-led model by investigating treatment effect, acceptability, and compare the results to previous findings. Treatment effect was defined as dropout, response rate, and effect size of change pre-post. Our results show that the total completion rate was high (78.0%), with a low dropout rate (11.0%) and only 11.0% step-ups during the active treatment phase (T1–T2). This completion rate was significantly higher than that reported by Salloum et al. (2022a, b), who had a dropout rate of 23.0%, but comparable to the one study including children in the same age range (Salloum et al., 2017).

Also, the response rate was comparable to previous findings. Two of three children enrolled in the study were not in need of more trauma treatment at the last assessment (T3), and almost four in five of those who completed the active treatment phase responded to the treatment. These results are encouraging, given that the mean baseline score of PTSS was above the clinical cutoff with 74.4% of the sample scoring above the cutoff for probable PTSD (Sachser et al., 2022), and the children reported exposure to significantly more traumatizing events compared to the two previous studies (Salloum et al., 2017, 2022a, b). In line with this, the treatment effect sizes were all large, both for reductions of PTSS (Cohen’s *d* = 2.46) and improvement of child functioning (Cohen’s *d* = 1.40). In sum, our findings show that the model was used with success in this new setting, and results are promising with regards to the potential dissemination of the model both to sites outside the U.S. and for children exposed to multiple traumas.

Assessments of parents’ satisfaction with the treatment is an important feature given that they are assigned partial responsibility for the completion of the treatment components. Since parenting practices are deeply rooted in culture (Lansford, 2022) it was not given that the parent-led model would have been accepted in a new setting without major adaptations. In particular, we expected that the idea of letting the caregivers lead the treatment, including the exposure tasks, themselves (instead of leaving this responsibility to an expert), the use of behavior plans, and mostly involving only one of the caregivers instead of both, would be met with some skepticism by the caregivers. However, what we found was that the caregivers reported high levels of satisfaction with both the quality and extent of the treatment, and the treatment outcomes. These results were comparable to the previous studies (Salloum et al., 2017, 2022a, b) and are also in line with results from qualitative studies of the model (Fagermoen et al., 2023a; Muster et al., 2022; Salloum et al., 2015a, b, 2016a, b). In the interview studies conducted by the developer, parents reported that they liked the parent–child meetings and that they found the Stepping Together workbook very helpful. In the current study (Fagermoen et al., 2023b) the interviewed parents highlighted that although this mode of treatment was new and challenging, being given the responsibility to lead the treatment was also very helpful as they gained a better understanding of their child’s problems. They also reported that they had learned new ways to meet their child that both improved their relationship and could be helpful if the child experiences new challenges in the future. Finally, the parents reported that they felt that the therapist had met their need to include the second caregiver when necessary.

The children also reported overall high levels of satisfaction (over 8 on a scale from 0–10). We cannot pinpoint from this scale what about the model they did or did not appreciate. However, previous studies have found that children like the relaxation exercises the most and many find the narrative and exposure components the most helpful. At the same time, the narrative/exposure component was also the least liked by the children (Salloum et al., 2015a, b). A more in-depth understanding of how the children find the model and their perceptions of having their parents in the role as the therapist, will be useful for future dissemination of the model.

The second aim of this study was to investigate child characteristics that could predict outcome, as this might help therapists better decide which children should start with the first parent-led step, and which should go directly to standard therapist-led treatment. Overall, our findings were mixed. We found that when we looked at the variables one and one, higher levels of PTSS, depressive symptoms and posttraumatic cognitions – and lower levels of externalizing behaviors – were associated with non-response after the active treatment phase (T2). However, predictor analyses showed that only PTSS and depression remained significantly associated with outcome post-treatment (T3) and that these results were no longer statistically significant when all variables were included in the adjusted model. These results are opposite of the study of Salloum et al. (2022b) who only found that severity of child’s anger outbursts predicted outcome, but neither baseline PTSS nor depressive symptoms. Due to the relatively modest sample size in both studies (*n* = 82 and 63 respectively), it is difficult to decide whether these differences reflect actual differences between the two cultural settings, or whether the results are to be interpreted as more random differences between these two specific samples. Therefore, firm conclusions about tailoring variables that can guide therapist decisions on which children should be offered a more therapist-intensive treatment from start cannot be drawn. Future studies with larger samples should investigate this further. However, from a clinical perspective, given that the model includes a close follow-up on the child’s improvement during treatment (with the CGI), it seems like a safe alternative to start with the parent-led treatment although we do not know enough about whom are more likely to respond and not. The promising results so far (Salloum et al., 2022a, b) indicate that the parent-led model can be used as a stand-alone treatment that may be a viable alternative if there is no access to therapist-led TF-CBT. Future studies should investigate whether there are additional benefits of the parent-led treatment (e.g., improvements in the parent–child relationship and strengthened parent-skills preventing the need for future treatment) that may make it worth completing the first step also for those families who are stepped up to receive additional therapist-led treatment or whether the parent-led treatment would rather represent a delay in their improvement. Also, including other potential tailoring variables such as parenting style, child-parent communication, strength of parent–child attachment or child and/or caregiver interpersonal problems may provide important new knowledge.

## Limitations

Although this study has several strengths, including being the first to investigate the model in a new context, several limitations warrant mentioning. First, this was a limited feasibility study with a relatively low sample size, thus results must be considered preliminary and should be confirmed in larger studies. Second, given the direct link between missing data at post-treatment and leaving the study due to treatment non-response, many of the children with poorer outcomes were not included in the results reported at mid-treatment and post-treatment. This should be considered when interpreting the reported group mean scores at mid- and post-treatment as the PTSS-scores may have been higher had follow-up data also been available for non-responders. Also, since the therapists were responsible for recruiting participants to the study, we cannot rule out that they may have tended to include families that were perceived as particularly positive or favorable for the treatment, resulting in a biased sample. Third, the relatively homogenous sample with only very few children from a non-Norwegian background limits the generalizability of our findings. Since background variables related to socioeconomic status are likely relevant for treatment outcomes, larger studies with a more heterogenous sample are needed to understand more of both child and caregiver tailoring variables.

## Conclusion

Overall, results from this study indicate that ST-CT can be delivered with success as a low-intensity stand-alone treatment in a new cultural context. Specific child characteristics such as initial symptom levels and exposure to interpersonal trauma may be related to the outcome of the ST-CT treatment, however this finding must be confirmed in larger and more heterogeneous samples. Although some children needed more therapist-intensive treatment, the majority improved sufficiently during the parent-led treatment and did not need further follow-up despite clinically significant levels of impairment before treatment. Also, although the model is manualized, the therapists were able to adapt the intervention to make it fit into this new cultural context and still deliver the treatment with fidelity. This may have important implications both at the individual and societal level. The parents that led their child’s treatment in the current study learnt new skills that may be useful in the eventual situation that their child is exposed to a new traumatizing event. At the societal level, partially shifting the treatment responsibility from a therapist to the parent means that therapist resources can be freed, allowing therapists to spend more time with families unable to complete the parent-led treatment. Future studies should investigate how to tailor the treatment even better to enable parents and children to complete the model, reduce barriers and enhance facilitators related to the successful implementation of the model in a new context.

## Data Availability

The data are not readily available due to the small and potentially identifiable dataset. Data may be made available upon request after the participant list has been deleted by the project leader.
